# Novel small-eye allele in paired box gene 6 (Pax6) is caused by a point mutation in intron 7 and creates a new exon

**Published:** 2013-04-12

**Authors:** Oliver Puk, Xiaohe Yan, Sibylle Sabrautzki, Helmut Fuchs, Valérie Gailus-Durner, Martin Hrabě de Angelis, Jochen Graw

**Affiliations:** 1 Helmholtz Center Munich, German Research Center for Environmental Health, German Mouse Clinic, Institute of Developmental Genetics, Neuherberg, Germany; 2 Helmholtz Center Munich, German Research Center for Environmental Health, Institute of Experimental Genetics, Neuherberg, Germany; 3 Helmholtz Center Munich, German Research Center for Environmental Health, German Mouse Clinic, Institute of Experimental Genetics, Neuherberg, Germany; 4 Chair of Experimental Genetics, Technische Universitat München, Center of Life and Food Sciences, Freising-Weihenstephan, Germany; 5 Helmholtz Center Munich, German Research Center for Environmental Health, Institute of Developmental Genetics, Neuherberg, Germany

## Abstract

**Purpose:**

Within a mutagenesis screen, we identified the new mouse mutant *Aey80* with small eyes; homozygous mutants were not obtained. The aim of the study was its molecular characterization.

**Methods:**

We analyzed the offspring of paternally *N*-ethyl-*N*-nitrosourea (ENU)–treated C3HeB/FeJ mice for dysmorphology parameters, which can be observed with the naked eye. The *Aey80* mutant (abnormality of the eye) was further characterized with laser interference biometry, Scheimpflug imaging, and optical coherence tomography. Linkage analysis of the *Aey80* mutant was performed using a panel of single nucleotide polymorphisms different among C3HeB/FeJ and C57BL/6J mice. The *Aey80* mutation was identified with sequence analysis of the positional candidate gene.

**Results:**

We identified a new mutant characterized by an obvious small-eye phenotype; homozygotes are not viable after birth. Embryos at embryonic day 15.5 demonstrate a clear gene-dosage effect: Heterozygotes have small eyes, whereas homozygous mutants do not have eyes. In adult mice, the lenses and the entire eyes of the heterozygous mutants were significantly smaller than those of the wild-types (p<0.01). No other ocular phenotypes were observed; the lenses were fully transparent, and no adhesion to the cornea was observed. The mutation was mapped to chromosome 2; markers between 70.8 MB and 129.5 MB showed significant linkage to the mutation resulting in paired box gene 6 (*Pax6*) as an excellent candidate gene. We amplified cDNAs from the embryonic eyes and observed an additional band while amplifying the region corresponding to exons 7 and 8. The additional band included an alternative exon of 141 bp, which was associated with a G->A exchange four bases downstream of the end of the alternative exon. The alternative exon in the mutants is predicted to encode 30 novel amino acids and three stop codons. This alternative exon kept the paired domain intact but led to a loss of the homeodomain and the C-terminal proline-serine-threonine (PST) domain. The mutation cosegregated in the mutant line, since all five additional small-eyed mice from this line showed the same mutation. A general polymorphism at the mutated site was excluded with sequence analysis of seven other wild-type mouse strains different from C3HeB/FeJ.

**Conclusions:**

These findings demonstrate a novel allele of the paired box gene 6 (*Pax6*) that affects lens development in a semidominant manner leading to a classical small-eye phenotype. However, the site of the mutation more than 1 kb downstream of exon 7 and resulting in an alternative exon is quite unusual. It indicates the importance of sequence analysis of cDNA for mutation detection; mutations like this are unlikely to be identified by analyzing genomic sequences only. Moreover, this particular mutation demonstrates how a novel exon can be created by only a single base-pair exchange.

## Introduction

Paired box gene 6 *(Pax6*) is the paradigm for a master control gene in eye development. This gene belongs to the family of genes that encode transcription factors with a homeodomain and a paired domain. Loss of *Pax6* function leads to the eyeless phenotype in *Drosophila*. Pioneering work by Walter Gehring's group in 1995 [1] showed that ectopic expression of the mouse *Pax6* induces functional ommatidial eyes in *Drosophila* in antennae or legs. This result suggested that at least from a genetic point of view, there is one way to make an eye. The first mouse mutation described in *Pax6* leads in heterozygous mutants to small eyes, but homozygous mutants have only remnants of ocular tissues and die shortly after birth because of nasal dysfunction [2]. Actually, in mice 38 distinct alleles have been described with different consequences for eye development (MGI database, Sept 2012). The most severe group has no (or almost no) Pax6 activity and includes the homozygous mutants *Pax6^3Neu^* and *Pax6^7Neu^*. On the other side of the allelic series are hypomorphic alleles like *Pax6^14Neu^*. In this mutant line (even if they are homozygous for the underlying mutation), all major eye tissues (cornea, lens, and retina) develop. However, eye size is reduced, and a large plug of persistent epithelial cells remains attached between the lens and the cornea (for a review, see [3] and references therein).

*Pax6* mutants are an example of mouse mutants that carry mutations in genes important in early stages of embryonic development and thus demonstrating pleiotropic effects including death of the homozygotes. These pleiotropic effects in most of the *Pax6* mutants include the brain with the olfactory bulb, development of the nose, and the pancreas. In the forebrain, Pax6 is critical for establishing the pallial-subpallial boundary, which separates dorsal (the future cerebral cortex) and ventral (the future striatum) telencephalic regions. Levels of *Pax6* expression are critically important for cortical progenitor proliferation, and the presence of *Pax6* in a rostrolateral (high) to caudomedial (low) gradient in the cortex is necessary to establish rostrolateral identities (for review, see [4]). Moreover, Pax6 is also important in various other developmental processes in the brain, including patterning of the neural tube, migration of neurons, formation of neural circuits, and in embryonic and postnatal neurogenesis. In particular, Pax6 is involved in producing new neurons from neural stem/progenitor cells, because it is intensely expressed throughout life in these cells from the initial stage of brain development and in neurogenic niches (the subgranular zone of the hippocampal dentate gyrus and in the subventricular zone of the lateral ventricle; for a review, see [5]). Moreover, Pax6 regulates survival of dopaminergic olfactory bulb neurons via α-crystallin [6].

Finally, Pax6 is crucial for endocrine cell differentiation and function. In several different *Pax6* alleles, we recently showed that Pax6 is also important for pancreatic α-cell development [7]. Moreover, mutations of *Pax6* are associated with a diabetic phenotype and a drastic decrease in the insulin-positive cell number. Recently, a Pax6-deficient model in rat primary β-cells was developed using a specific small interfering RNA leading to a 75% knockdown of *Pax6* expression [8]. These authors demonstrated that Pax6 controls the mRNA levels of several target genes, including the genes encoding insulin 1 and 2; the authors also demonstrated that *Pax6* knockdown led to decreases in insulin cell content and insulin processing, and a specific defect of glucose-induced insulin secretion in primary β-cells.

In humans, *PAX6* mutations mainly cause aniridia, a panocular disorder, and less commonly isolated cataracts, macular hypoplasia, keratitis, and Peter’s anomaly (for a recent review, see [9] and references therein), microphthalmia [10,11], and microcornea in rare cases [12]. In the mouse, homozygous loss of *Pax6* function affects all expressing tissues and is neonatal lethal [13,14]. Therefore, it might be of medical interest that *Pax6* is expressed not only in the optic field and in the lens but also in several brain regions and in the pancreas. Correspondingly, there is a growing body of evidence that *PAX6* mutations cause, in addition to ocular diseases, behavioral and neurodevelopmental phenotypes as well as disorders of the pancreas [15-17]. The actual *PAX6* database contains more than 345 entries of unique human variations of *PAX6* (Feb. 2013), which might also result in a clinical heterogeneity.

Here we report on a novel *Pax6* allele leading to a classical small-eye phenotype. However, the mutation occurred more than 1 kb downstream of exon 7 and results in an alternative exon. This is quite unusual and unexpected; it also indicates the importance of sequence analysis of cDNA for mutation detection, because mutations such as this are unlikely to be identified by analyzing genomic sequences only. Moreover, this particular mutation demonstrates how a novel exon can be created with only a single base-pair exchange. Mechanisms such as this are of exceptional importance for understanding evolutionary processes.

## Methods

### Mice

Mice were kept under specific pathogen-free conditions at the Helmholtz Center Munich. The use of animals was in accordance with the German Law of Animal Protection, the Association for Research in Vision and Ophthalmology Statement for the Use of Animals in Ophthalmic and Vision Research, and the tenets of the Declaration of Helsinki and approved by the Government of Upper Bavaria under registration number 55.2–1- 54–2531–78–06. Male C3HeB/FeJ mice were treated with *N*-ethyl-*N*-nitrosourea (ENU; 90 mg/kg body weight applied by intraperitoneal injection in three weekly intervals) at the age of 10–12 weeks as previously described [18] and mated to untreated female C3HeB/FeJ mice [19]. The offspring of the ENU-treated mice were screened at the age of 11 weeks for dysmorphology parameters.

### Eye morphology and function

For histologic analysis mice were killed by cervical translocation and, the heads of the embryos were fixed for 3 h in Carnoy’s solution and embedded in JB-4 plastic medium (Polysciences Inc., Eppelheim, Germany) according to the manufacturer’s protocol. Sectioning was performed with an ultramicrotome (OMU3; Reichert-Jung, Walldorf, Germany). Serial transverse 3-μm sections were cut with a glass knife and stained with methylene blue and basic fuchsin as described previously [20]. The eyes of the *Aey80* mutant mice were evaluated at 19 weeks of age. For laser interference biometry and optical coherence tomography, mice were anaesthetized with 137 mg ketamine and 6.6 mg xylazine per kg bodyweight. Eyes were further treated with 1% atropine to ensure pupil dilation.

Eye size measurement was performed using the “AC Master” (Meditec, Carl Zeiss, Jena, Germany). Briefly, anaesthetized mice were placed on a platform and oriented in an appropriate position using light signals from six infrared light-emitting diods (LEDs) arranged in a circle that must be placed in the center of the pupil. Central measurements of axial eye length were performed essentially as described [21].

Eye fundus and retina were analyzed with a Spectralis OCT (Heidelberg Engineering, Heidelberg, Germany) modified with a 78 diopter double aspheric lens (Volk Optical, Inc., Mentor, OH) fixed directly to the outlet of the device. To the eye of the mouse, a contact lens with a focal length of 10 mm (Roland Consult, Brandenburg, Germany) was applied with a drop of methyl cellulose (Methocel 2%, OmniVision, Puchheim, Germany). For measurements, anaesthetized mice were placed on a platform in front of the Spectralis OCT such that the eye was directly facing the lens of the recording unit. Images were taken as described previously [22]. Retinal thickness was calculated with the provided thickness profile tool.

Images of corneas and lenses were taken with the Pentacam digital camera system (Oculus GmbH, Wetzlar, Germany). Mice were held on a platform in a way that the vertical light slit (light source: LEDs, 475 nm) was oriented in the middle of the eye ball. The distance between the eye and the camera was finely adjusted with the help of the provided software to guarantee optimal focus. Subsequently, measurements were started manually. Mean density across the lens was quantified with the provided densitometry tool. For statistical evaluation, medians, first and third quartiles, and p values were calculated with a Wilcoxon rank-sum test. Statistical significance was set at p<0.05.

### Linkage analysis

Heterozygous carriers (first generation) were mated to wild-type C57BL/6J mice, and the offspring (second generation) were again backcrossed to wild-type C57BL/6J mice. DNA was prepared from the tail tips of the affected offspring of the third generation (G3). For linkage analysis, genotyping of a genome-wide mapping panel consisting of 153 single nucleotide polymorphisms (SNPs) was performed using MassExtend, a matrix-assisted laser/desorption ionization, time of flight (MALDI-TOF) analyzer mass spectrometry high-throughput genotyping system supplied by Sequenom (San Diego, CA) [23].

### Genotyping and sequencing

RNA was isolated from embryonic mouse eyes (E15.5) and reverse transcribed to cDNA using the T-Primed First-Strand kit (Amersham Bioscience/GE-Health Care, Freiburg, Germany). Genomic DNA was isolated from the tail tips of 102, 129, Balb/c, C3HeB/FeJ, C57BL/6J, CBA, DBA/2J, and JF1 wild-type mice or homozygous/heterozygous embryos (E15.5; on C3HeB/FeJ background) according to standard procedures. PCR was performed with a Flex Cycler (Analytik Jena, Jena, Germany) using primers and conditions listed in Table 1. Products were analyzed with electrophoresis on a 1.5% agarose gel. Sequencing was performed commercially (GATC Biotech, Konstanz, Germany) after direct purification of the PCR products (Nucleospin Extract II, Macherey- Nagel, Düren, Germany). To confirm the mutation in the genomic DNA, a 320-bp fragment was amplified from genomic DNA using the primer pairs Pax6-intron7-L1 and –R1 (Table 1).

**Table 1:** List of PCR primers

Lab-No	Sequence (5′ ->3′)	Fragment size	Annealing temperature
Pax6-L11	CTCACAGGCAGAAGACTTTAACC^1^	682 bp	53–64 °C^3^
Pax6-R11	CTTCCTGTTGCTGGCAGC^1^		
Pax6-L12	CAACCTGGCTAGCGAAAAGC^1^	648 bp	53–64 °C^3^
Pax6-R12	TGCATAGGCAGGTTGTTTGC^1^		
Pax6-L13	CTATCAGCAGCAGCTTCAGTACC^1^	616 bp	53–64 °C^3^
Pax6-R13	TTGTTCCAACTGATACCGTGC^1^		
Pax6-Ex7-Int7-L1	ACAGAGTTCTTCGCAACCTGGC^2^	320 bp	67 °C^4^
Pax6-Ex7-Int7-R1	CCTTGACATACATAATCCTTACAGTCACC^2^		
Pax6-Intron7-L2	TGTGAATCGGTGAGCTCTTAGACC^2^	410 bp	67 °C^4^
Pax6-Intron7-R2	TACATCAGAAGCCTGCACTGACC^2^		

^1^the primers for PCR on cDNA are based upon the reference sequence BC036957.1 (GenBank); ^2^the primers for PCR on genomic DNA are based upon ENSMUST000000111086 (ENSEMBL); ^3^PCR was performed using Phusion-DNA Polymerase (Fermentas); ^4^PCR was performed using DreamTaq Polymerase (Fermentas).

### General

Chemicals and enzymes were from Fermentas (St-Leon-Rot, Germany), Merck (Darmstadt, Germany), or Sigma Chemicals (Deisenhofen, Germany). Oligonucleotides were synthesized by Sigma Genosys (Steinheim, Germany).

## Results and Discussion

**Figure 1:**
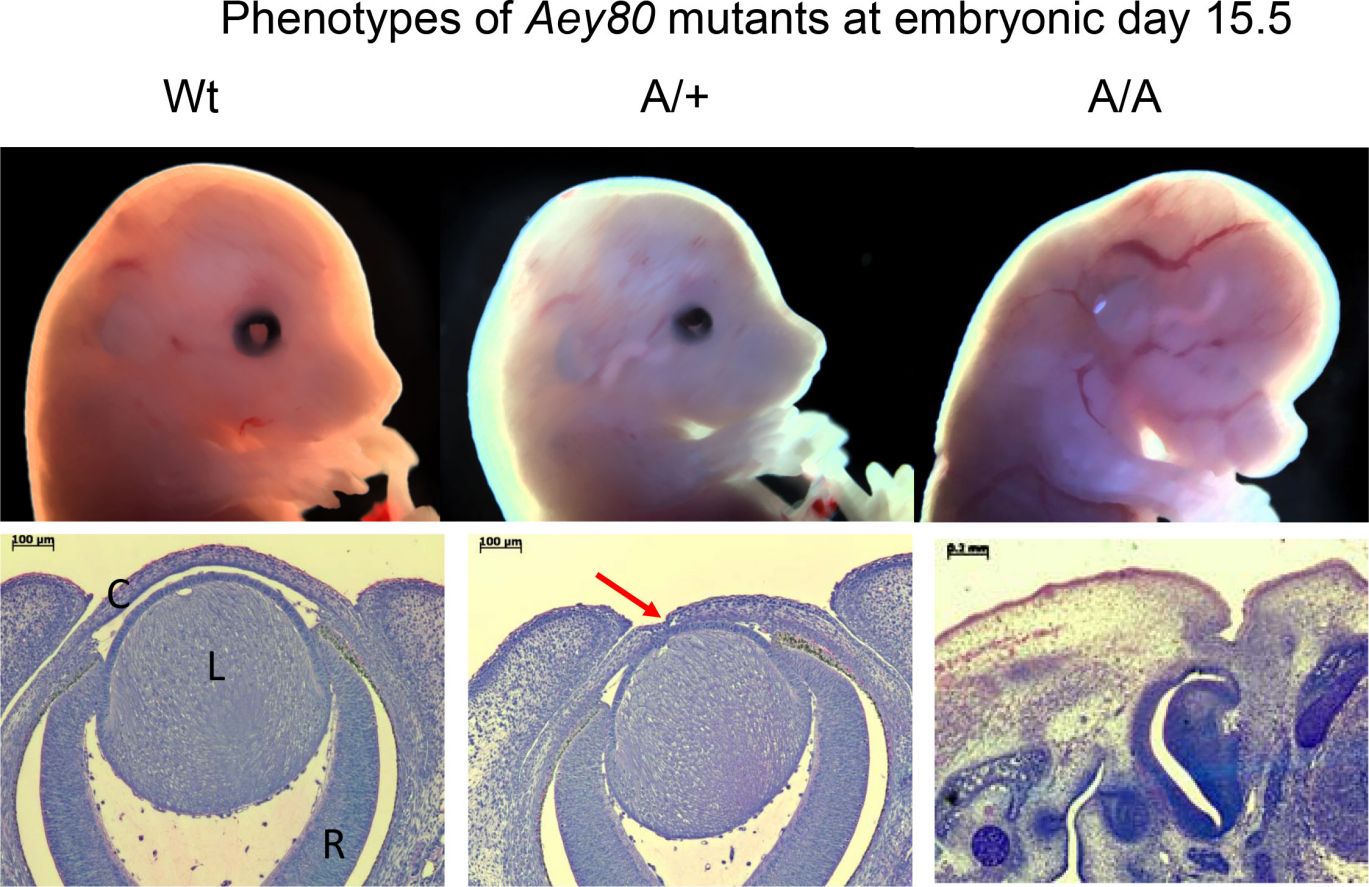
Phenotypes of *Aey80* mutants at embryonic day 15.5. Upper row: Littermates of the Aey80 mutant line show at embryonic day 15.5 three different phenotypes: regular eye size in wild-type mice (left, WT), a smaller eye in heterozygous (middle), and embryos without any eyes in homozygous mutants (right). These figures are similar to those described previously for *Pax6* mutant embryos [20,26]. Lower row: Histological section through the eye: wild-type eye (left) with regular cornea (C), lens (L) and retina (R); heterozygotes frequently exhibit a synechia between lens and cornea (middle, red arrow); homozygous mutants do not show ocular structures.

Offspring from ENU-treated male mice were screened for different phenotypic parameters including general dysmorphology. The mutant *Aey80* (abnormality of the eye) was picked up because of its small-eye phenotype. During breeding, no homozygous mutants were obtained; however, during embryogenesis, three different phenotypes can be observed (Figure 1) suggesting that the homozygous phenotype (without eyes) does not survive after birth.

**Figure 2:**
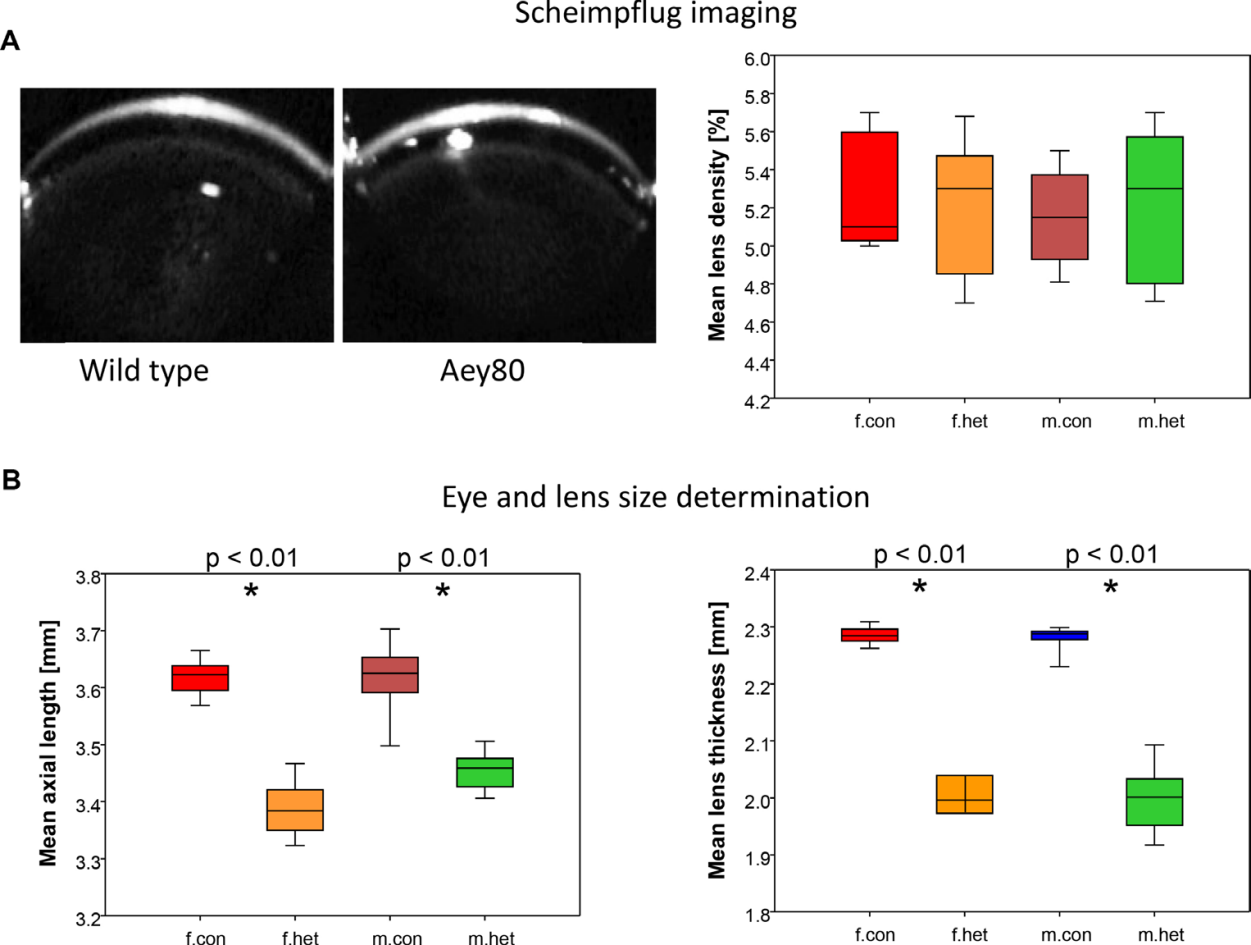
Ocular phenotyping of *Aey80* mutants. **A**: Scheimpflug analysis demonstrates clear lenses without lens-cornea adhesions in adult wild-type and heterozygous *Aey80* mutants (left: typical images). Right: boxplots of the mean lens density (%) of the right eyes; the data for the left-eye lenses are similar. **B**: The eyes (axial length; left) and the lenses (right) of the mutants (males and females) are significantly smaller in the heterozygous *Aey80* mutants compared to the wild-types (p<0.01). The colored boxes indicate half of the data (between the 0.25 and 0.75 quantile); the end of the vertical lines indicate the lowest and highest values, respectively; the bars in the colored boxes indicate the median. n=10 in each group; f, females; m, males; con, wild-type; het, heterozygous *Aey80* mutants.

The eyes of the adult mice were extensively analyzed within the framework of the German mouse clinic [24]. Scheimpflug analysis demonstrated clear cornea and lenses (Figure 2A); the cornea-lens adhesion observed in the embryonic mutants was no longer present in the adult mice. Since the mutant appeared on the C3H genetic background, *Aey80* mutant mice are blind due to the retinal degeneration based upon the C3H-specific *Pde6b^rd1^* mutation [25]. The only differences observed were significantly smaller lenses in the mutants resulting in the smaller size of the entire eye (Figure 2B).

**Figure 3:**
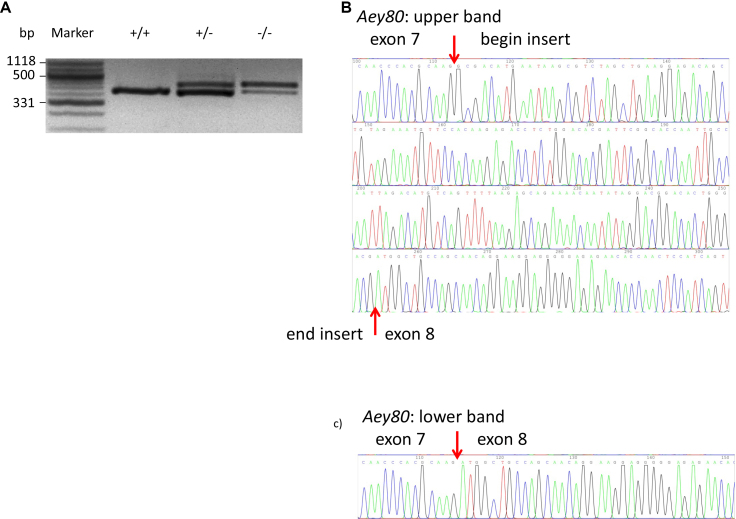
Analysis of *Aey80* cDNA. **A**: Amplification of the middle part of the *Pax6* cDNA revealed an additional higher band in the *Aey80* mutants. **B**: Sequence analysis of the larger fragment demonstrated an insert of 141 bp between the exons 7 and 8. **C**: This insert is not present in the lower (wild-type) band.

**Figure 4:**
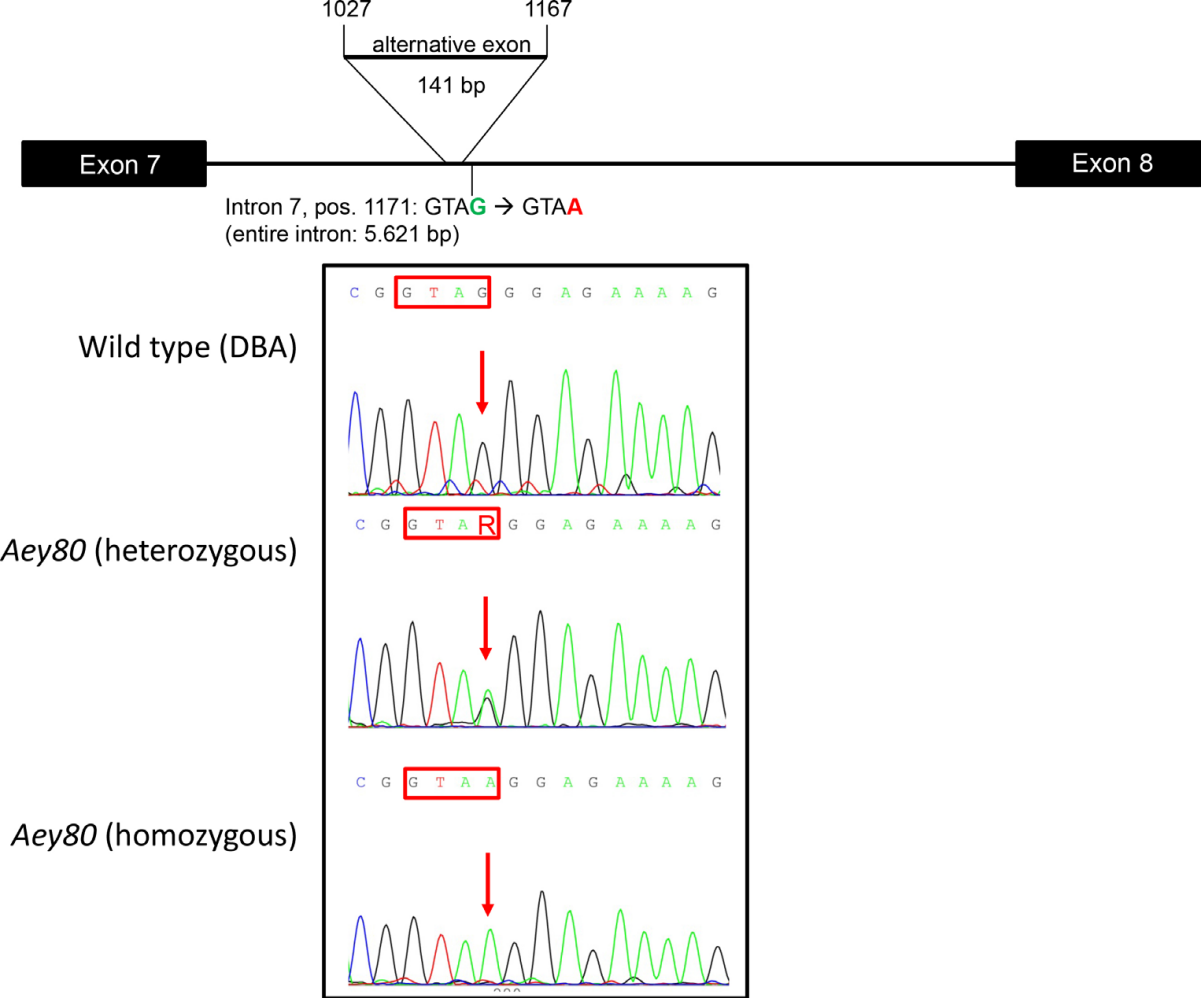
Analysis of *Aey80* genomic DNA. Sequence analysis of Pax6 intron 7 identified the 141 bp to be flanked by a G->T exchange four bases downstream of the insert sequence. Upper part: schematic drawing of the region of exons 7 and 8 including the intronic region; the location of the insert is indicated (not drawn to scale). Lower part: The four bases downstream of the new insert are given and particularly boxed in the sequences. Heterozygosity (R: G and A) is obvious in the heterozygous mutants; homozygous *Aey80* mutants (embryos only) are given in the third row of sequences.

In a genome-wide linkage analysis using SNP markers, the mutation was mapped to chromosome 2; markers within the interval between 70.8 MB and 129.5 MB showed a significant linkage to the mutated phenotype. Together with the phenotype observed, this linkage analysis made *Pax6* to a promising candidate gene for the underlying mutation. Amplifying *Pax6* cDNA derived from heterozygous embryos resulted in an extra band, if the middle part of the *Pax6* cDNA was amplified (Figure 3A). Sequencing of the corresponding fragments identified an insert of 141 bp between the regular exons 7 and 8 (Figure 3B,C). The alternatively spliced exon of 141 bp corresponded to a part of intron 7 spanning in total 5.621 bp. Sequencing of the corresponding region (410 bp) of *Pax6*-intron 7 demonstrated a G->A exchange in the sequence of the mutant, which was located 4 bp downstream of the novel alternatively spliced exon (Figure 4). This mutation cosegregated within the breeding colony (five mutant mice tested) but did not represent a general polymorphism since the mutation was not present in wild-type mice of different strains (102, 129, Balb/c, C3HeB/FeJ, C57BL/6J, CBA, DBA/2J, and JF1). After 186 amino acids of the wild-type Pax6, this novel alternative exon led to the formation of 30 new amino acids followed by three stop codons within 36 bp. Therefore, this mutation left the paired-box domain intact; however, the homeodomain was not formed.

Applying a splice-site prediction program (SplicePredictor) to this sequence, it is immediately obvious that the mutant sequence has a high probability (>90%) to form a new exon. At position 1026 of the intron, a likely splice acceptor site was present, but without a corresponding splice donor site. However, the mutation at position 1171 created such a splice-donor site with a similar probability resulting in the formation of this new exon as observed in the mutant mice. The formation of this new exon by the exchange of a single base pair is an excellent example of exonization as an evolutionary process, even if it is deleterious in this particular case.

The mutation in the *Aey80* mutants disrupted the *Pax6* gene between its paired domain and homeobox leading to the classical “small-eye” phenotype as it was originally described [2], a small eye in the heterozygotes and no eye in the homozygous embryos, which were not viable after birth. Similar features have been described previously for other *Pax6* alleles affecting the same linker region such as the *Pax6^3Neu^* allele [26] and the *Pax6^Aey11^* and *Pax6^ADD4802^* alleles [27]. Actually, the MGI database covers in total 38 *Pax6* alleles of different origin, and eight other cell lines come from gene trap or related resources. However, comparison of the biologic effects of this allelic series demonstrates a broad variety of severity among the *Pax6* alleles leading to hyper- and hypomorphic lines (most examples are given in [28]). In summary, mouse and human data demonstrate that *Pax6*/*PAX6* is frequently targeted by mutations leading to a broad variety of phenotypes and clinical manifestation.
